# Supplementary material for: Learning curve and surgical outcome of robotic assisted colorectal surgery with ERAS program
Source: Sci Rep. 2022 Nov 29;12:20566. doi: 10.1038/s41598-022-24665-w (PMC9709162; doi:10.1038/s41598-022-24665-w)
Supplement: Supplementary file 2 — Supplementary Information 2. [file 41598_2022_24665_MOESM2_ESM.zip › RAL Raw data-Ñ[▒K/062/OP N_ñw╝╨░O▒Kñσ.pdf]

W52 / W52

臺中榮民總醫院

手術室護理記錄

[redacted]

基本資料

- 室別： 開刀房-17室，手術科別： CRS，手術類別： 常規，日期： 2021/02/25
- 進入時間： 1255 ； 麻醉開始時間： 1300 ； Time Out時間： 1415 ； 劃刀時間： 1415 ； 手術結束時間： 1635 送出時間： 1655 ；
- 麻醉方式： GE

  診斷： Malignant tumor of sigmoid colon

  手術名稱： Robot Assisted Colectomy(L)

  手術局部用藥： NIL

  術前預防性抗生素：
    - 抗生素名稱一： Veterin 1GM IVD 由麻醉護理人員協助執行
    - 時間： 1355
    - 上次簽章: 蘇敬涵

  ，追加預防性抗生素：
- 手術醫師： 蔣鋒帆，楊曙榮，楊立宇
- 麻醉醫師： VS 張思玲
-
    - 刷手護士A ： 邱文琇，時間： 1255，刷手護士B ： 廖子晴，時間： 1255，接替刷手一： 羅媗鈴，時間： 1500
    - 流動護士A ： 蘇敬涵，時間： 1255，接替流動一： 紀玉娟，時間： 1500

皮膚狀況

A-V Shunt： 無

術前： 完整

記錄者： 上次簽章: 蘇敬涵

術後： 不完整

部位(術後)：

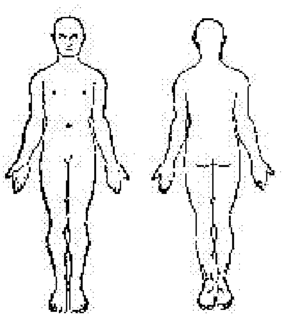

右眼4*0.5公分破皮及左眼4*0.5公分破皮(3M)，下巴2*2公分發紅不可白。

記錄者： 紀玉娟

手術準備

手術臥式 單一手術臥式

-
    - 手術臥式： 截石臥式
    - 使用附件： 溫毯 38 ℃，頭圈，其它： lateral support
    - 皮膚準備： 10%Aq Betadine，Hibiscrub，2% Chlorhexidine
    - 電燒： 使用電燒: 電極片位置： 右大腿，記錄者： 上次簽章: 蘇敬涵

止血帶T： 無

植入物

- 植入物名稱/號碼/數量/部位： Da Vinci Xi Endo Wrist Stapler 45*4.3mm*2/S colon

- stapler circular 29mm/S colon

, 記錄者： 上次簽章: 紀玉娟

標本送檢

- 細菌培養 無
- 細胞學 無
- 冰凍切片 無
- 病理標本
  組織名稱一： IMA root
  ,
  組織名稱二： distal cut end
  ,
  組織名稱三： S-colon
- 截肢 無

標本交付 標本： 1 袋 ， 標本： 0 盒 ， 標本： 2 瓶 ， 細胞學： 0 管 ， Culture Tube： 0 管 ， 被交付醫師： R 楊立宇 ，

- 記錄者： 上次簽章: 紀玉娟
- 時間： 1620

傷口術後敷料

傷口敷料： 傷口敷料： 無

- 請於圖上標示部位：

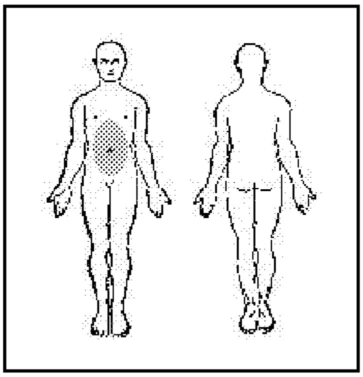

- 傷口敷料： 軟性敷料

導管

導管	天數	導管資訊	插入日期	插入時間	更換日期	拔除日期	拔除時間	拔除原因	預計拔除/更換	預計拔除/更換日期
Epidural PCA(1)	1	部位:硬脊膜外;	2021/02/25	1355						
Double Lumen CVC(1)	1	號碼:8 Fr.; 深度:16cm; 部位:jugular (右); 象限:RU;	2021/02/25	1355						
Foley(1)	1	號碼:16 Fr.; 部位:膀胱;	2021/02/25	1355						
A-line (1)	1	號碼:20 G; 部位:左手;	2021/02/25	1355						

用物計數

- 手術中出血量： minimal c.c.
- 手術中尿量： 600 c.c.
-
  - 其它： 無

用物計數： 多切口同時縫合

- 1.器械： 正確 ， 2.縫針： 正確 ， 3.刀片： 正確 ， 4.紗布： 正確 ， 5.腹部墊： 未使用 ， 6.紗球： 未使用 ， 7.PEANUTS ： 未使用 ， 8.紗條： 未使用 ， 9.絲帶： 未使用 ， 10.針頭： 未使用 ， 11.Vessel loop： 未使用 ， 12.Boots： 未使用 ， 13.Jelco： 未使用 ， 14.棉條： 未使用 ，
  - 第一次：
    - 刷手護士 ： 羅媗鈴
    - 流動護士： 上次簽章: 紀玉娟

- 時間： 1550

,
- 第二次：
  - 刷手護士 ： 羅媗鈴
  - 流動護士： 上次簽章: 紀玉娟
  - 時間： 1600

,
- 第三次：
  - 刷手護士 ： 羅媗鈴
  - 流動護士： 上次簽章: 紀玉娟
  - 時間： 1610

備註

備註： Da Vinci Scope NO.1

- 記錄者： 上次簽章: 蘇敬涵
- 時間： 1311

填寫護理人員 紀玉娟

正式摘要

W52 / W52

doctorRank:B
